# Study on the Extraction of CT Images with Non-Uniform Illumination for the Microstructure of Asphalt Mixture

**DOI:** 10.3390/ma15207364

**Published:** 2022-10-20

**Authors:** Lei Zhang, Guiping Zheng, Kai Zhang, Yongfeng Wang, Changming Chen, Liting Zhao, Jiquan Xu, Xinqing Liu, Liqing Wang, Yiqiu Tan, Chao Xing

**Affiliations:** 1 School of Transportation Science and Engineering, Harbin Institute of Technology, Harbin 150090, China; 2 China State Construction International Holdings Limited, Hong Kong 999077, China; 3 CCCC NO. 1 Highway Survey Design & Research Institute Co., Ltd., Xi’an 710065, China; 4 Sichuan Gezhouba Batongwan Expressway Co., Ltd., Bazhong 636600, China

**Keywords:** asphalt mixture, Computed Tomography, image process, microstructure, gradation analysis

## Abstract

An adaptive image-processing method for CT images of asphalt mixture is proposed in this paper. Different methods are compared according to the error analysis calculated between the real gradation and 3D reconstruction gradation. As revealed by the test results, the adaptive image-processing method was effective in carrying out different brightness homogenization processes for each image. The Wiener filter with 7 × 7 size filter was able to produce a better noise reduction effect without compromising image sharpness. Among the three methods, the adaptive image-processing method performed best in the accuracy of coarse aggregate recognition, followed by the ring division method and the global threshold segmentation method. The error of the gradation extracted by the adaptive image-processing method was found to be lowest compared with the real gradation. For a variety of engineering applications, the developed method helps to improve the analysis of CT images of asphalt mixtures.

## 1. Introduction

As variable multi-phase composite material, asphalt mixture is characterized by significant differences, randomness, and variability among the constituent materials, which contributes to the non-uniqueness of its microstructure. However, the spatial distribution characteristics of each component in the internal structure of asphalt mixture cannot be reflected by the traditional macro-empirical evaluation method, which focuses primarily on the overall features and road performance of asphalt mixture at the macro scale. However, for multi-phase asphalt mixture, the macro mechanical properties depend on its micro-structures. In this circumstance, it is necessary to calculate the performance mechanism of asphalt mixture from a meso-level perspective. At present, computed tomography (CT) and digital image-processing technology have been widely applied in micro-scale research on asphalt mixture. It is difficult to properly identify the differential materials in CT images of asphalt mixture, due to factors such as CT imaging mechanism and variations of asphalt-mixture gradation composition. Meanwhile, grayscale values of substances with the same density should theoretically be consistent at different locations, but the scanning energy limitation of industrial CT machines causes them to present varying degrees of lightness and darkness at different distances from the specimen’s central axis. Therefore, it remains challenging to accurately identify aggregates, voids, and asphalt mortar. To obtain an accurate extraction of the microstructure of asphalt mixture, it is necessary to further study the image-processing method used for CT images of asphalt mixture.

It was in the early 1990s that research began on the CT image-processing method used for asphalt mixture. At that time, Masad et al. [1] applied CT technology and digital camera technology for the first time to conduct a quantitative analysis of the compaction effect of the mixture under different compaction modes, which led to success in introducing CT into the research on the microstructure of asphalt mixture, thus opening up a new field of research. In the meantime, research was advanced on the image-processing method applied for composite CT.

CT scanning technology is used to detect the attenuation of X-rays after passing through an object. Then, the attenuation images are outputted to the reconstruction algorithm for completing the reconstruction [2]. Due to the poly-chromatic nature of X-ray energy, however, the attenuation coefficient can cause errors, thus resulting in artifacts within the reconstructed images [3,4]. The methods used to correct artifacts can be categorized into physical filtering methods and software correction methods. Commonly used during scanning, the physical filtering method [5,6,7,8] can reduce the artifacts. Meanwhile, however, it also reduces ray intensity, which contributes to SNR (signal noise ratio) reduction. Due to fewer restrictions during application, software correction methods [3,9,10,11] are also frequently used. For asphalt mixture, however, which is a multiphase material, these methods are more complicated for correcting artifacts due to the lack of prior knowledge about X-ray spectra and material properties.

In recent years, CT scanning technology has been increasingly used for the microstructural characterization of asphalt [12], bituminous mortar [13], and asphalt mixture. Within research on asphalt-mixture CT image processing, the focus has mainly been placed on the methods emloyed to separate aggregate, mortar, and void within the image [14,15]. Currently, commonly used methods include the artificial threshold method [16,17,18], fuzzy C-means algorithm [19,20], Gaussian mixture model [21], and Otsu algorithm [22,23,24,25]. The most widely used is the Otsu algorithm. When Otsu is applied to images with artifacts, however, the segmentation effect is less than satisfactory. To solve this problem, Liu put forward the ring division method [26], which achieved higher segmentation accuracy. Nevertheless, it failed to determine accurately the size of the test piece and its position in the image. Additionally, the method was restricted to reducing the impact of artifacts on image segmentation when faced with the artifacts distributed along the radial non-linear section of the test piece, meaning it was unable completely to address the problem of artifacts.

In order to address the current shortcomings of image-processing methods, this paper proposes a brightening program for asphalt-mixture CT images that can help effectively eliminate the adverse effect of artifacts on image threshold segmentation. The processing effect based on this method was compared with that produced by the ring division method and the global threshold segmentation method, respectively. The research findings of this paper can contribute to more precisely identifying the composition and different materials of asphalt mixtures, accurately ascertaining the microstructure of asphalt mixture, and accomplishing a quantitative analysis of void distribution characteristics, aggregate homogeneity, and microscopic damage characteristics of asphalt mixture. This can provide a thorough understanding of the internal microstructure and properties of asphalt mixture, and improve the relevant test methods and protocols to further improve the performance of pavements. In addition, the method can also be applied to cement concrete, composite materials, and metal materials, so it has promising application potential.

## 2. Materials and Methods

### 2.1. Materials

In this study, andesite produced in Heilongjiang Province and limestone powder produced in Jilin Province were used. The relevant tests were conducted on stone materials, in line with the standards JTGE42-2005 [27] and JTGF40-2004 [28]. According to the test results, all indicators met the requirements. SBS polymer modified asphalt was used in this study, and the properties are shown in Table 1. The lignin fiber content of the SMA asphalt mixture was 0.3%.

### 2.2. Gradation Design

**Figure 1:**
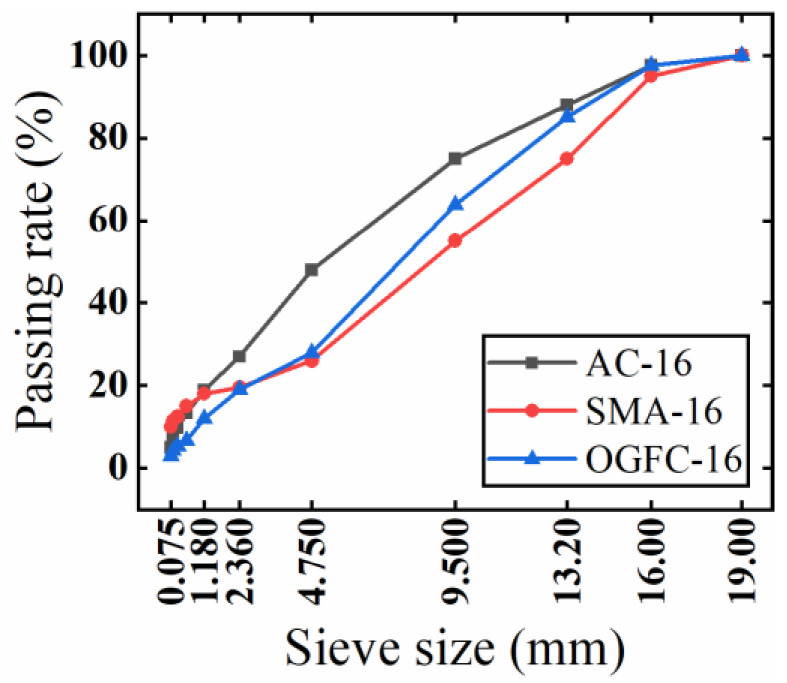
Gradation curves of three kinds of asphalt mixtures.

AC-16, OGFC-16, and SMA-16 test pieces were designed and fabricated according to the standard JTGF40-2004, with three replicates prepared for each sample. The gradation is shown below in Figure 1.

### 2.3. Optimum Asphalt Content and Test Pieces Preparation

The optimum oil content of the asphalt mixture was determined using the Marshall asphalt mixture design method. The optimum asphalt content of AC-16, SMA-16, and OGFC-16 was 4.5%, 6%, and 3.1%, respectively. The mixtures were compacted 120 times using the rotary compaction method. To obtain more pictures of the test pieces, the height of the test pieces after molding was set to about 150 mm. The volume parameters of the asphalt mixtures are shown in Table 2.

### 2.4. Scan by Industrial CT

The CT scanning equipment used for Marshall test pieces was a Phoenix micro focus industrial CT purchased by the School of Transportation Science and Engineering affiliated to Harbin Institute of Technology. The cone-beam scanning industrial CT system and the cone-beam filter back-projection reconstruction algorithm were both applied to reconstruct the faulty images. The scanning voltage/current was 195 kv/95 μA. The critical performance indicators are shown in Table 3. In this study, 150 mm corresponded to 1500 pixels in the image, so the resolution of the asphalt-mixture image recognition was 0.1 mm.

For the original model subjected to CT scanning and 3D reconstruction, the cross-sectional images were exported at equal spacing, with the spacing value set to 0.15 mm, and each test piece was capable of exporting a total of 1000 images.

## 3. CT Image Feature Analysis

**Figure 2:**
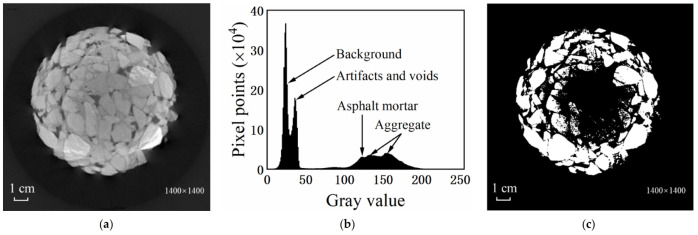
Characteristics of CT images. (**a**) Original image; (**b**) gray histogram; (**c**) global segmentation.

Taking SMA-16 as an example, there were roughly five peaks shown in the gray histogram of the asphalt-mixture CT image. Under normal conditions, each peak is represented in ascending order of gray scale: background, voids and artifacts, asphalt mortar, and aggregates. In practice, however, the artifacts appeared in the center of the test piece within the image, i.e., the brightness near the center was lower than near the edge, and the brightness along the radius of the test piece was nonlinear, which is largely attributed to ray hardening. Consequently, the gray value of some aggregate shifted to the wave peak of mortar. In case of artifacts being left unprocessed, the aggregate was extracted directly using the global threshold segmentation, causing the aggregate within the artifacts to be recognized as background removal, thus resulting in the loss of aggregate information as shown in Figure 2. This is where the difficulty lies when processing asphalt-mixture CT images.

## 4. Image-Processing Method for Asphalt Mixture

### 4.1. Adaptive CT Image-Processing Method

#### 4.1.1. Obtain the Center and Radius of the Test Piece

It is important to determine the center and radius of the test piece for subsequent image-processing operations. During the actual CT scanning process, however, there is no way to guarantee that the test piece is located at the center of the scanning platform. Thus, the test piece can show different center positions in the images. Therefore, it is necessary to identify the center and radius of the test piece in the image.

Firstly, the matrix of the entire image was extracted into a rectangular coordinate system, with the row and column numbers of the pixel points representing their Y and X coordinates respectively.

**Figure 3:**
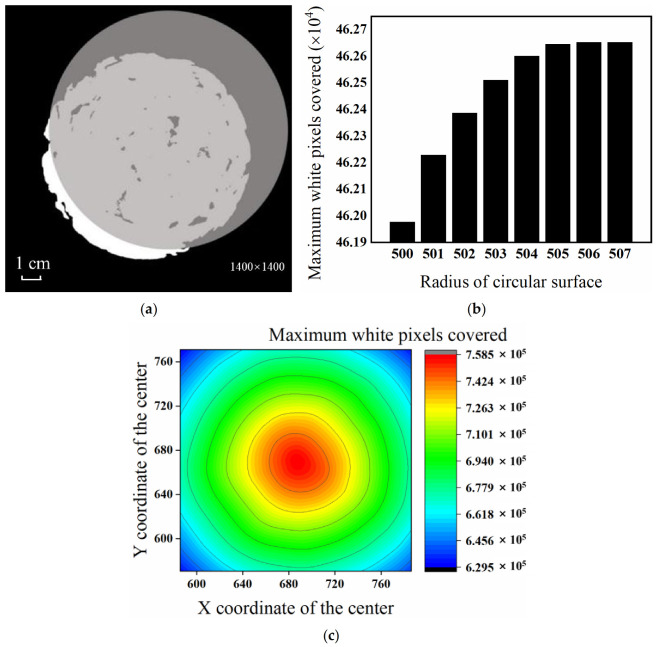
Diagram of center and radius’s determination. (**a**) Circular surface covering white pixels; (**b**) determined radius of the test piece; (**c**) determined center of the test piece.

Then, the image was segmented using a single threshold. A range of values was set for the radius of the circle surface is, starting from the minimum value, and a circular surface with that radius value was drawn and allowed to move freely over the binarized image. As shown in Figure 3, the maximum number of white pixels it can cover was recorded, and then the radius value was increased by one pixel length. The above steps were repeated until the radius of the circular surface reached the maximum value, or the maximum number of white pixels covered after increasing the radius value by one pixel length remained unchanged. At that time, the iteration process was discontinued.

Initially, following the method described above, the upper limit on the radius of the circular surface was excessively small. The upper limit was increased and iteration continued. In the second case, the maximum number of white pixels that can be covered by the circular surface with the value of adjacent radius was identical, suggesting that the smaller value was the radius of the test piece. Then, the circular surface was drawn with the radius of the test piece, so that it could be moved within the image. When it covered the maximum number of white pixels, the center of the circular surface overlapped with the center of the test piece, meaning that the center of the test piece could be determined.

Finally, the radius and center of test piece were used for segmenting the image to remove the redundant information while improving the processing efficiency.

#### 4.1.2. Image Brightness Homogenization

Since the distribution of the artifacts was a circular range concentric with the test piece, the brightness adjustment operation was conducted by calculating the distribution of the grayscale of the test piece along the radius direction, with the procedures detailed as follows.

**Figure 4:**
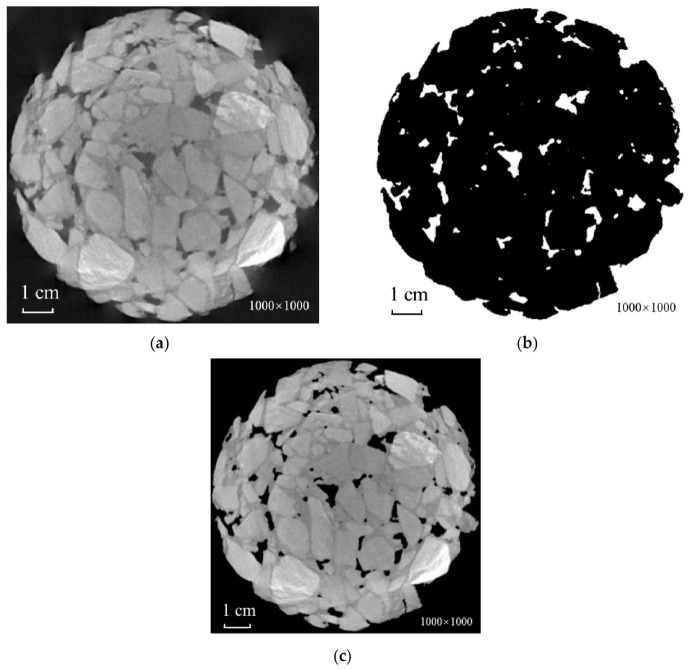
Zero processing of grayscale voids. (**a**) Cropped image; (**b**) extraction of voids (white part); (**c**) voids’ gray value return to zero.

**Figure 5:**
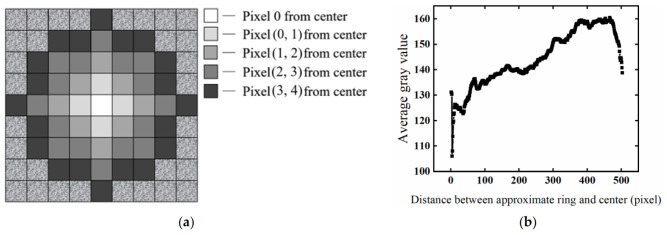
Obtained gray distribution along the radial direction. (**a**) Approximate ring concentric with the test piece; (**b**) radial distribution of grayscale.

(1) Zero processing of the grayscale of voids. Since the extracted object used in this study was aggregated, it was necessary for mortar and voids to be removed as background. However, mortar cannot be removed due to the presence of artifacts, resulting in the grayscale of mortar and making the aggregates overlap each other. In order to prevent this from impacting the calculation of the statistical distribution of grayscale along the radius, multi-threshold segmentation and image subtraction [22] were first performed to remove the voids, as shown in Figure 4.

(2) Calculating the distribution of grayscale along the radial direction. Based on the previously determined center of the test piece, the average gray values of the pixels on the approximate ring concentric with the test piece were calculated one by one. The expression of each approximate ring is shown in (1), while the approximate ring is shown in Figure 5a.

(1) $$i-1<\sqrt{\left(x-x_{0}\right)+\left(y-y_{0}\right)}\leq i$$

where *x*_0_ and *y*_0_ represent the horizontal and vertical coordinates of the center of the test piece, respectively; *x* and *y* indicate the horizontal and vertical coordinates of the pixel points on the *i*-th approximate ring, respectively; *i* denotes the outer diameter of the approximate ring, and the value ranges from one pixel length to radius.

After determining the approximate ring that different pixels belong to, the average gray value of the pixels on each approximate ring was calculated, as shown in Formula (2).

(2) $$Average_{i}=Sum_{i}/Num_{i}$$

where *Sum_i_* represents the sum of the pixels’ gray value with the gray value of [0, 180] on the *i*-th approximation ring; *Num_i_* indicates the number of pixels with the gray value of [0, 180] on the *i*-th approximation ring; and *Average_i_* denotes the average gray value of pixels with the gray value of [0, 180] on the *i*-th approximation ring.

If the radius of the test piece is 503 pixels in length, 503 average values can be calculated. To avoid affecting the brightening operation, inclusion was limited to pixels with gray values greater than 0 but less than 180 before the average value was calculated. This is because the aggregates with large gray value and voids did not need to be increased in brightness, so they were excluded from the calculation. Figure 5b shows the radial distribution of gray value.

(3) Based on the average gray value of the pixels in each approximate ring, 180 was taken as the target gray value. When the average was no greater than 180, the gray value of the pixels on the *i*-th approximate ring was processed as follows, and the pixels in the whole image whose gray value exceeded 180 were made equal to 180:

(3) $$gray_{x,y}=gray_{x,y}+180-Average_{i}$$

where *gray_x,y_* represents the pixels whose gray value was less than 180 on the *i*-th approximate ring; *x, y* indicate their horizontal and vertical coordinates; and 180 is the target gray value.

The one-dimensional entropy of an image reflects the average amount of information contained in the image, and provides the aggregation characteristics of gray distribution for the image. The larger the entropy is, the greater the probability of different levels of gray in the image. One-dimensional entropy of the image was introduced to evaluate the brightness homogenization process. The formula is:

(4) $$H=-\sum_{0}^{255}p_{i}\times \log_{2}p_{i}$$

where *H* represents the image entropy and *p_i_* indicates the probability of the *i*-th gray value appearing in the image, obtainable from the gray histogram.

The results of image entropy calculation before and after processing were between 5.336 and 4.869. The entropy value of images after brightness homogenization was shown to be significantly lower than before processing, suggesting that the gray value of images after processing was more uniform and the processing effect was satisfactory.

**Figure 6:**
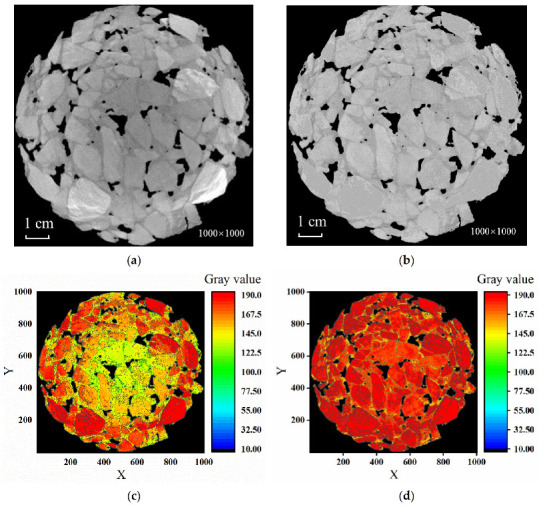
Diagram of the effect of luminance homogenization. (**a**) Original image; (**b**) image after brightness homogenization; (**c**) grayscale contour map of original image; (**d**) after brightness homogenization.

The problem of artifacts was effectively reduced after the image had been processed by brightness homogenization as shown in Figure 6. Additionally, the differences in grayscale between the central region and the edge region of the test piece were reduced significantly, and the brightness of the image was more uniform, which facilitated the threshold segmentation of the image.

#### 4.1.3. Image Filtering and Noise Reduction

**Figure 7:**
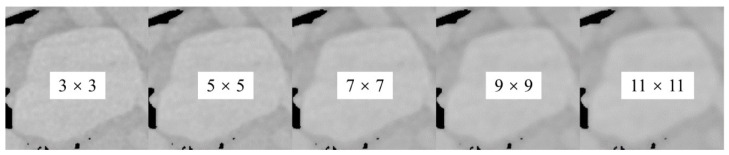
Comparison of filtering effects using filters of different sizes.

The existence of noise makes the threshold segmentation program prone to errors in the recognition of aggregate edge, and can cause adverse effects on the calculation of threshold, as a result of which the threshold segmentation program shows sensitivity to noise. It was thus necessary to filter the image for the purpose of noise reduction. In this study, the adaptive low-pass Wiener filter [29] was applied to filter the grayscale images, with 3 × 3, 5 × 5, 7 × 7, 9 × 9, 11 × 11 selected respectively as filter windows, to estimate the mean and standard deviation of local images, shown in Figure 7.

It can be seen in the figure that the amount of noise around the aggregate particles diminished with the increase of the filter window. Furthermore, the gray value of aggregate became more uniform. Meanwhile, however, the application of a larger filter caused severe loss of image details, thus resulting in deterioration of image clarity and aggregate edge identification. In this study, 7 × 7 filter window was applied to reduce the image noise.

**Figure 8:**
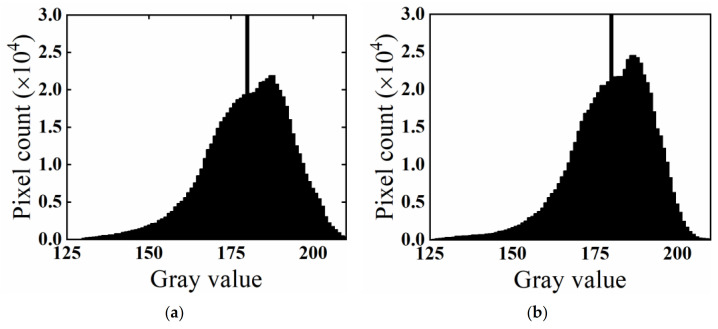
Diagram of noise reduction effect. (**a**) Before noise reduction; (**b**) after noise reduction.

The effect of the 7 × 7 filter is shown in Figure 8, revealing that the peak of the representative aggregate in the gray histogram narrowed but shifted upward after noise reduction, indicating that the aggregate gray value tended to be uniform and that the effect of noise reduction was evident.

#### 4.1.4. Global Otsu Threshold Segmentation

**Figure 9:**
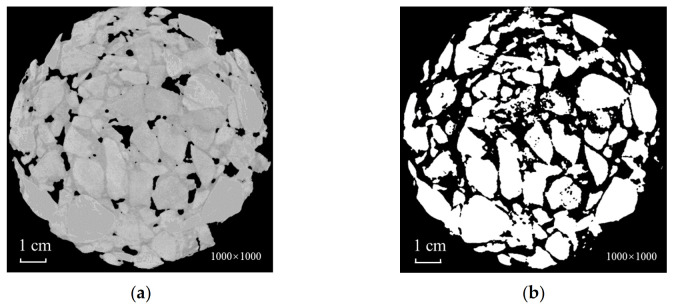
Diagram of the adaptive processing method’s threshold segmentation. (**a**) Before threshold segmentation; (**b**) after threshold segmentation.

Depending on the grayscale characteristics of the image, the Otsu algorithm divides the image into two parts: background and foreground, with the variance taken as a measurement of the uniformity of grayscale distribution. Thus, the greater the variance between the background and foreground, the more significant are the differences between the two parts of the image. In this situation, the threshold calculated by the Otsu algorithm is considered best when the variance between the two parts is the largest. Therefore, Otsu thresholding based on the whole image was selected to perform binarization. At this stage, the gray value of voids and artifacts in the image was returned to zero, and the image contained as few as three parts, i.e., black background, asphalt mortar, and aggregate. The aim of binarization is to separate the aggregate. Therefore, in this study, Otsu was applied to calculate the double threshold. The smaller threshold was the boundary between the background and the asphalt mortar, while the larger threshold was the boundary between asphalt mortar and aggregate. The higher threshold was used for segmentation, with the gray value of mortar restored to zero. The result is shown in Figure 9.

#### 4.1.5. Image Morphological Processing

After thresholding segmentation, the holes require filling and conglutinated aggregates need to be separated. The holes distributed in the image can be classed into two categories. One results from the uneven texture of the aggregate and causes the low grayscale after CT scanning. This can be eliminated by the algorithm, and holes of this kind need to be filled. The other category relates to the proximity between the two adjacent aggregate edges, where the gaps between them become holes after threshold segmentation, and should be retained. This lays a foundation for the subsequent separation of the adhered aggregates by the watershed segmentation algorithm.

**Figure 10:**
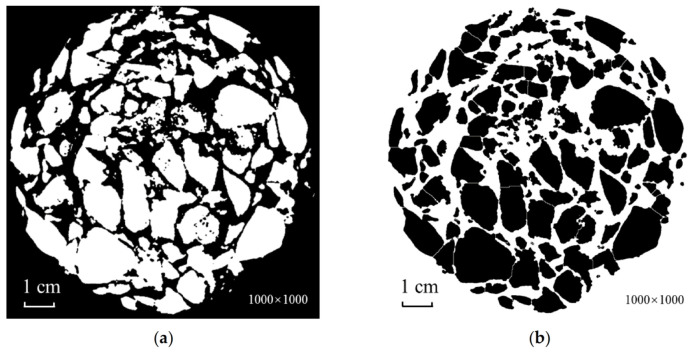
Comparison of before and after morphological treatment. (**a**) Before morphological processing; (**b**) after morphological processing.

In this study, the image was first processed in reverse color, and then eroded [22], as a result of which the adjacent aggregate was separated. In the meantime, the holes inside the aggregates expanded, while remaining closed holes. The closed holes were filled, the holes inside the aggregates were removed, and the image was dilated to restore the size of the aggregates. Finally, the watershed algorithm [17,18,30] was applied to separate further the adhered aggregates. The outcome of the treatment is shown in Figure 10.

### 4.2. Ring Division Method

With regard to the CT image processing of asphalt mixture, it has been proposed by some scholars that the mixture can be divided into multiple ring blocks, and that the impact of artifacts on threshold segmentation can be mitigated by threshold segmentation being performed in each part of the image. The final image can be obtained by combining the images after binary processing. The core idea is that the artifacts are distributed in a non-linear way along the radial direction of the test piece. In general, if segmentation based on the global threshold is adopted directly, the closer the gray value of the aggregate to the center of the test piece, the lower is the gray value difference. A more significant gray value difference in the aggregate will contribute to the removal of the aggregate from the center of the test piece. If the image is divided into ring blocks, however, the difference in the aggregate’s gray level in each part of the image can be reduced, while the precision of threshold segmentation can be improved.

**Figure 11:**
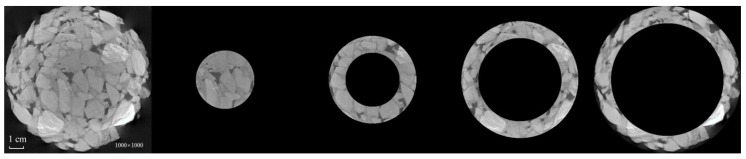
Partition of original image.

**Figure 12:**
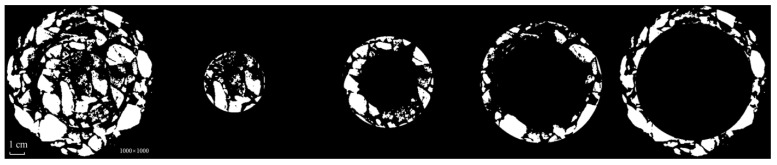
Diagram of the image-processing method based on ring division.

The asphalt-mixture CT image was split into three rings and one circular surface, in line with the ring division rule, with each part overlapping each other. Each part of the image was filtered and denoised, the threshold was segmented, and the combined image was morphologically processed, as shown in Figure 11 and Figure 12, respectively.

It was found that the CT image-processing method based on ring division was effective for mitigating the impact of artifacts on threshold segmentation, by splitting rings. The loss of aggregate recognition was less severe than with global threshold segmentation, and boundaries were clearly seen when the binary image was combined with a complete image. Compared with the adaptive CT image-processing method, an evident gap was observed in the completeness of the aggregate recognition.

## 5. Grading Verification of 3D Reconstruction Model

**Figure 13:**
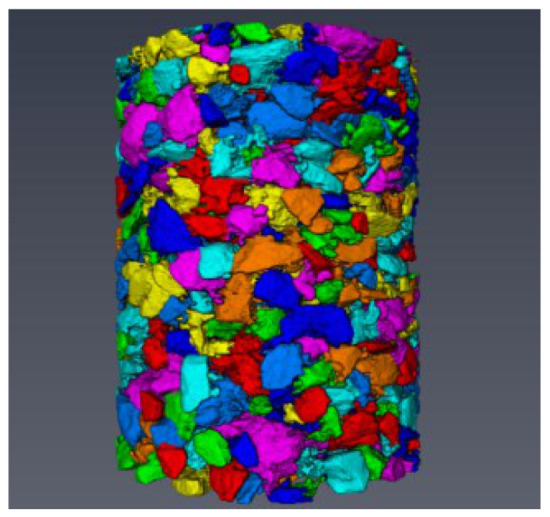
Three-dimensional reconstruction model of asphalt mixture.

To validate the image-processing method, the images processed using an adaptive image-processing method, the ring division method, and a global threshold segmentation method, respectively, were imported into Avizo software for 3D reconstruction [31,32]. The model is shown in Figure 13.

**Figure 14:**
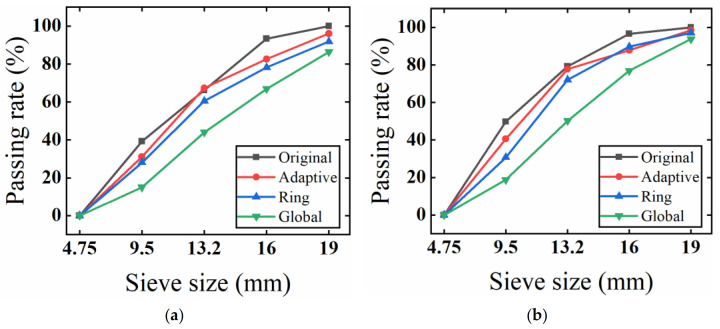

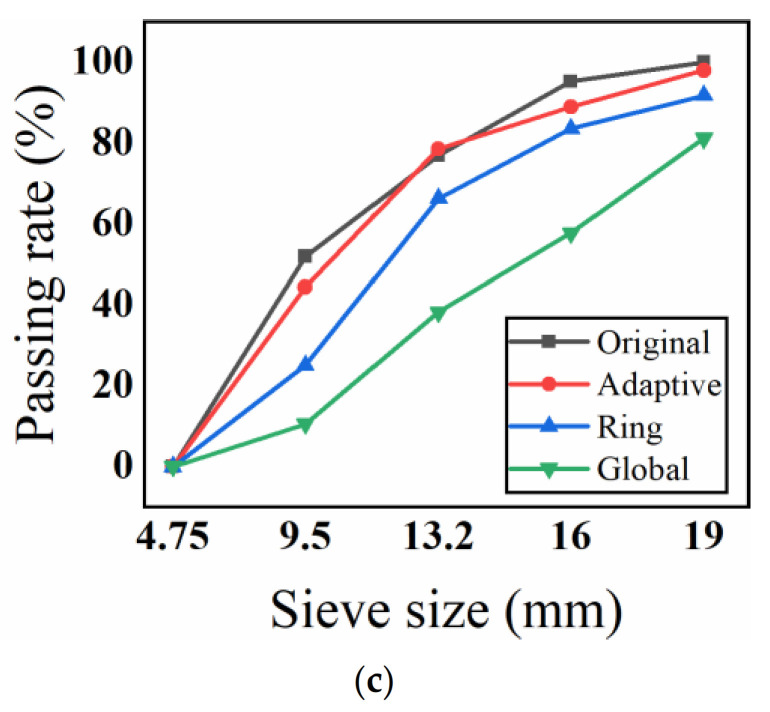
Comparison of three test pieces’ gradation curves. Adaptive: image processing based on the adaptive image-processing method. Ring: image processing based on ring division. Global: image processing based on global segmentation. (**a**) SMA grading comparison; (**b**) OGFC grading comparison; (**c**) AC grading comparison.

Applying the extracted volume information for the particles in the model, the particles were regarded as spheres, and virtual sieving was conducted. For the virtual sieving, the software automatically identified the volume of the particles and equate each particle with a sphere of the same volume. The diameter of the sphere was considered to be the diameter of the particle. Because the quality of the CT images was not as high as required, the accuracy of aggregate extraction below 4.75 mm was far from satisfactory. Furthermore, it was difficult for some of the fine aggregate to be separated by the algorithm, due to its close contact and adhesion with coarse aggregates. Its small area caused some fine aggregates to be eroded. Consequently, when the grading curve was drawn, it was suitable only for counting aggregates with a diameter of 4.75 mm or over. The true grading required conversion to a diameter of 4.75 mm or more. The comparison of gradation is shown in Figure 14. When model gradation was evaluated, it was also possible to regard the model gradation as a regression fitting to the true gradation, and to use statistical parameters to evaluate the regression fitting effect.

As shown in Figure 14, it was found that the three-dimensional reconstruction model established from the images processed with the adaptive method was the closest to the real gradation. The error analysis is shown in Table 4. The error of the model using the adaptive method was smaller compared with the method based on ring division, and significantly smaller compared with the method based on global threshold segmentation, suggesting that the adaptive method is more accurate than other methods for the identification and segmentation of aggregates. Thus, it can restore the true gradation of the aggregates to the greatest extent. It was also revealed that there was little difference in accuracy of aggregate recognition between different kinds of asphalt mixtures, which suggests that asphalt mixtures of different kinds have good adaptability to the application of CT images.

## 6. Conclusions

(1) The adaptive CT image-processing method was based on the recognition of the center and radius of the test piece in the image. After the voids were removed from the image, the gray distribution was counted along the radial direction of the test piece, the grayscale distribution along the radial direction of the test piece was adjusted to homogenize the brightness, and the one-dimensional entropy value of the image was applied to characterize the effect of brightness homogenization. As indicated by the results, the entropy value reduced significantly after the image was brightened. The problem of artifacts in CT images of asphalt mixture has been effectively resolved.
(2) The larger the window of the Wiener filter, the more significant was the noise reduction effect on the CT image. Simultaneously, however, it caused image sharpness to be reduced. The 7 × 7 Wiener filter was able to produce a better effect of noise reduction without compromising image sharpness.
(3) In order to avoid subjectivity caused by the visual observation of the image processing effect, the binary images obtained by the three methods were imported into the 3D reconstruction software to extract the particle information contained in the reconstruction model and to perform virtual sieving. The model’s gradation curve of aggregates of 4.75 mm and above was obtained, and the errors between the model gradation curves and the real gradation curves were characterized by statistical parameters. It was discovered that compared with other methods, model gradation caused the least significant error from the real gradation, the degree of aggregate adhesion was also lower, and the adaptive image-processing method showed a strong adaptability to the processing of CT images for different kinds of mixtures.

## Figures and Tables

**Table 1:** Properties of SBS modified asphalt.

Properties	Unit	Test Result	Specification Requirements	Specification
Penetration (25 °C, 100 g, 5 s)	0.1 mm	66.9	60~80	JTG F40
Ductility (5 °C, 5 cm/min)	cm	43.3	≥30	
Softening point	°C	66.5	≥55	

**Table 2:** Volume parameters of asphalt mixtures.

Mixtures	Air Voids Content (%)	VMA (%)	VFA (%)
AC-16	3.5	13.9	74.9
SMA-16	3.6	17.0	78.9
OGFC-16	19.7	28.2	30.2

**Table 3:** Key performance indicators of Phoenix micro-focus industrial CT.

Maximum Tube Voltage/kV	Maximum Tube Power/kW	Detail Resolution/μm	Minimum Distance from Focus to Test Pieces/mm	Maximum Pixel Resolution (3D)/μm	Geometric Magnification (2D)	Geometric Magnification (3D)
240	320	1	4.5	≤2	1.460–180	1.46–100

**Table 4:** Gradation error analysis.

Evaluation Parameter	SMA-16			OGFC-16			AC-16		
	Adaptive	Ring	Global	Adaptive	Ring	Global	Adaptive	Ring	Global
Absolute error	4.757	8.019	17.275	4.151	7.178	17.195	3.497	11.477	27.296

## Data Availability

All the data available in main text.
